# Bone impaction grafting with trabecular metal augments in large defects in young patients: unravelling a new perspective in surgical technique

**DOI:** 10.1186/s12891-020-03591-w

**Published:** 2020-08-27

**Authors:** Basilio De la Torre-Escuredo, Eva Gómez-García, Salvador Álvarez-Villar, Julia Bujan, Miguel A. Ortega

**Affiliations:** 1 Service of Traumatology of University Hospital Ramón y Cajal, Madrid, Spain; 2 Department of Surgery, Medical and Social Sciences, Faculty of Medicine and Health Sciences, University of Alcalá, Alcalá de Henares, Madrid, Spain; 3 Ramón y Cajal Institute of Sanitary Research (IRYCIS), Madrid, Spain; 4 Departments of Medicine and Medical Specialities, Faculty of Medicine and Health Sciences, University of Alcalá, Alcalá de Henares, Madrid, Spain

**Keywords:** Bone impaction grafting, Trabecular metal augments, Large acetabular defects, Young revision patients

## Abstract

**Background:**

Acetabular reconstruction with bone impaction grafting in large defects has yielded conflicting results.

**Methods:**

This was a retrospective study of a case series of five patients with a young age (≤50 years) at the time of surgery who had large acetabular defects reconstructed by bone impaction grafting and trabecular metal augments. The mean follow-up was 79 months. We describe the surgical technique in detail.

**Results:**

Improvement was significant on the WOMAC and SF-36 scales (*p* < 0.05). The radiographs taken at the last follow-up examination showed no migration of the polyethylene cup (*p* = 0.31) or differences in the abduction angle (*p* = 0.27) compared to the radiographs from the immediate postoperative period. One patient presented two dislocation episodes as a complication.

**Conclusion:**

The combination of trabecular metal augments with the bone impaction grafting technique in young patients with large acetabular defects provides satisfactory results in the long term and restores the bone stock.

## Background

Acetabular revision surgery for large defects is a challenge for orthopaedic surgeons. This challenge is greater when these defects occur in young patients, in whom it is essential to restore the centre of rotation of the hip, achieve stable implant fixation, and restore the acetabular integrity and the bone remnant.

Several treatment options have been described to achieve this end, with different results [1–6]. The only option that can restore bone remnant is bone allograft reconstruction. This allograft may be in structural form, with uncertain results in the literature [4, 7], or in the form of an impacted graft [8], a technique developed by the Nijmegen group [9].

Results with the latter type of reconstruction are good and reproducible in small defects, i.e., cavitary defects or segmental defects affecting less than 50% of the acetabular cavity [10, 11]. However, when the defect affects more than 50% of the acetabular cavity, the results are discouraging [12–14]. For this reason, we have proposed using trabecular metal (TM) augments in combination with bone impaction grafting to aid in the reconstruction of large bone defects. The high porosity of this material and its high coefficient of friction provide good mechanical stability [15].

The purpose of this study is to report the long-term results of this reconstruction method in a group of young patients (≤50 years old) at the time of surgery and with large acetabular defects.

## Patients and methods

The study identified a consecutive series of patients who underwent acetabular revision using trabecular metal augments and the bone impaction grafting technique during the period from May 2011 to May 2014. This time period was chosen to obtain a minimum follow-up of 5 years. During the study period, seven patients were operated on with this technique, but only five were included in the study, all of whom were under 50 years of age at the time of the intervention. The two excluded patients were 64 and 60 years old at the time of surgery.

**Fig. 1:**
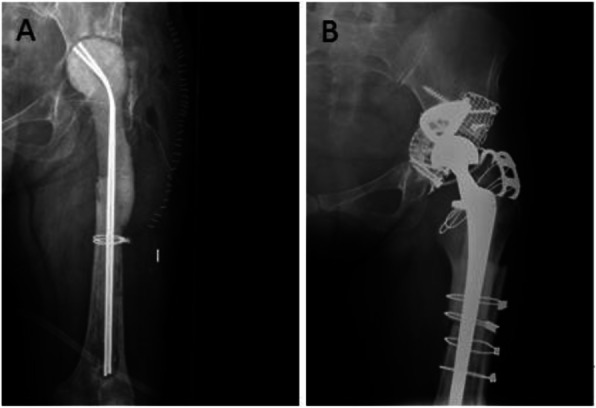
**a-b**. Preoperative radiograph (first stage) in a patient with septic loosening of the acetabular component and the last radiograph after reconstruction (case 2)

All patients were operated on by the author of the study, and none of them were lost to follow-up. The mean age of the patients was 46.8 years (45–50) at the time of the intervention, with a mean follow-up of 79 months (60–101). There were three women and two men. The initial diagnosis in two patients was osteoarthritis secondary to hip dysplasia, and in the other three, avascular necrosis of the femoral head (Table 1). In the two patients with a history of hip dysplasia, reconstruction was performed in two stages because both had septic acetabular loosening (Fig. 1). The only comorbidity was rheumatoid arthritis in a 47-year-old male (case 3) under treatment with biologics.

**Table 1:**
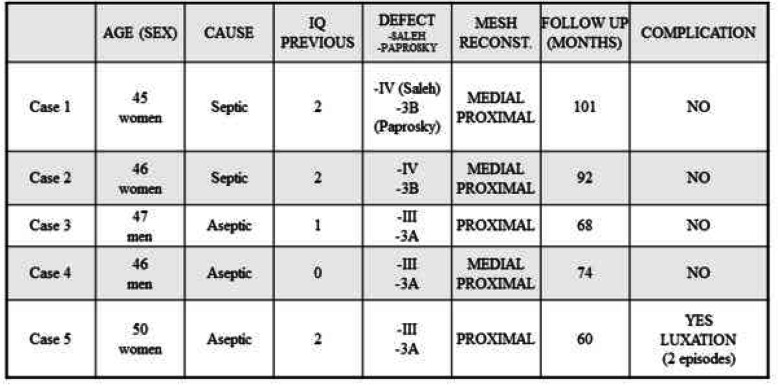
Patient demographic characteristics, indications for surgery, acetabular bone defects, follow-up times, and complications

Bone defects were classified according to the Paprosky classification [16] because it is the most widely used in the literature and the Saleh classification [17] because it is a simple, reliable, valid system for the classification of bone defects [18].

### Surgical technique

Patients were operated on in the lateral decubitus position using a posterolateral approach to the hip. In all cases, infection was ruled out before surgery through analytical studies. Arthrocentesis was also performed before capsulotomy for collection of a joint fluid sample. At this time, antibiotic prophylaxis was initiated according to our hospital protocol. Samples were taken from the acetabular fundus for both anatomopathological and microbiological studies. Once the pathologist reported the absence or ≤ 5% per field of polymorphonuclear leukocytes, the acetabular cavity reconstruction was started. Debridement of fibrous tissue was initiated by milling the acetabular remnant, and the bone defect was identified, with special attention to the posterior column, acetabular fundus and posterosuperior area. When the defect affected almost the entire posterior wall, we decided to perform another reconstruction method with acetabular augments and components made of trabecular metal, associated or not with a cage. In these cases, it was difficult to place the screws in the posterior column that supported the mesh in the posterosuperior portion to reconstruct the defect and achieve rigid fixation, and this is one of the most important aspects of the bone impaction grafting surgical technique. If the defect was in the fundus of the acetabulum (central defects), we placed a mesh in it (central mesh) that was affixed to the pelvis with at least three 3.5-mm screws. The ischiopubic notch (teardrop), when present, was used as a reference to reconstruct the centre of rotation to its anatomical position. In case of a defect in this area, we used the ischium as a reference for reconstruction. A trial acetabular reamer was placed in the anatomical position, and we again checked the defect in the posterosuperior zone. At this time, the trial TM augments were sized in the defect to allow sufficient space for impaction to take place. When we found the correct size, a trial reamer was placed in anatomical position and held to offer inferior support to the augment to avoid displacement during the fixation of the augment to the iliac bone with al least two 6.5-mm screws. Next, we placed the mesh in position to convert the uncontained defect into a contained defect. In these large defects, it was essential to affix the first screw to the ischial area and then the rest of the screws to the anterior column. Correct fixation of the anterior and posterior corners is essential. We checked to ensure rigid fixation of the mesh to the iliac bone.

Bone chips were prepared (fresh-frozen bone chips) by hand with a gouge clamp, obtaining sizes of 7–10 mm^3^ to provide graft stability. We pressure-washed the graft with a spray gun, and the allograft was then packed into the defects and into the TM spaces. Further layers of graft were impacted using impactors of different sizes. We started with the smallest impactor and continued with impactors of greater diameter, reconstructing the socket to the desired position. The last impactor used had a diameter 4 mm greater than the outer diameter of the polyethylene component to allow for an adequate cement mantle. At this moment, the bed of impacted graft should feel like a cortical bone. An all-polyethylene flanged cup was inserted into viscous cement, held in position, and held with pressure until the cement had polymerised. In one case (case 2) we used a Trident® constrained acetabular insert because we used a structural allograft to reconstruct the femoral side. This component was cemented in a position with lesser abduction angle.

Patients were mobilized with partial weight bearing during 12 weeks. 6 weeks with two crutches followed by transition to one crutch during 6 weeks, and full weight bearing as tolerated over following 4 weeks. By 4 months, patients were allowed full weight bearing.

Clinical outcome was assessed according to preoperative questionnaires and the last follow-up. Hip status was assessed using the Western Ontario and McMaster Universities (WOMAC) questionnaire [19]. Its usefulness comes from its ability to assess clinical changes patients have perceived in their state of health, and it has been validated in Spanish [20]. The numeric rating scale is scored from 0 (best) to 10 (worst). Therefore, the maximal aggregate score for pain, stiffness, and function is 50, 20, and 170, respectively. Generic health was assessed using the SF-36 [21], with its eight scales ranging from 0 to 100 (100 being best). The SF-36 also includes a transition item that asks about the change in general health status from the previous year. Lastly, every patient was asked whether they would undergo the operation again.

**Fig. 2:**
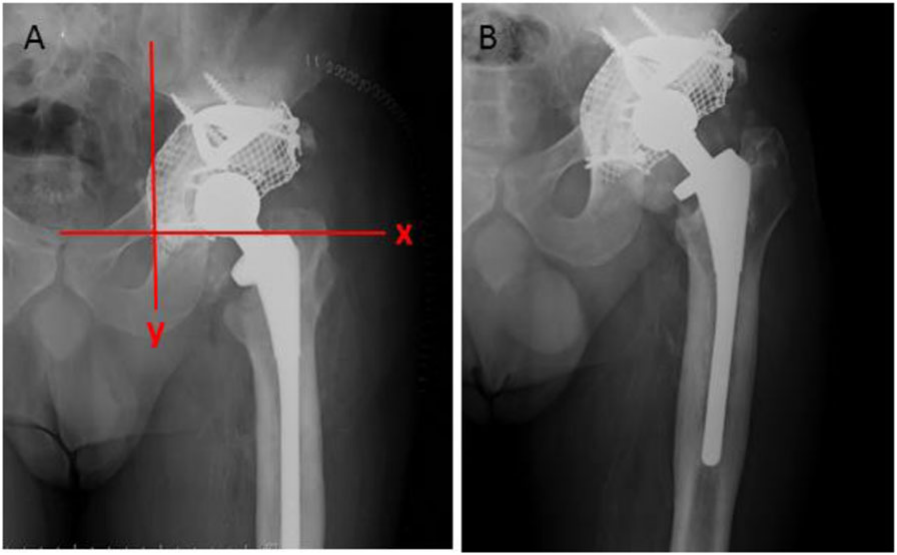
Case 4: (**a**) Radiographs immediately postoperatively and 6 years post-surgery (**b**) showing the acetabular component position using the method described

Radiological analysis was based on an anteroposterior (AP) radiograph of the pelvis and an axial projection taken preoperatively, immediately postoperatively, and at the last follow-up examination. Preoperative images gave an idea of the acetabular defect, but in all cases the defect was classified according to what was seen during surgery. To determine whether there was migration of the acetabular component, we established references on AP radiographs of the pelvis, following the study by Borland et al. [22]. A line was marked that joined the two acetabular teardrops (x-axis), and another line was perpendicular to the first lateral to the teardrop (y-axis). The horizontal and vertical distances to the polyethylene cup were measured at its lowest and medial points. We also measured the abduction angle of the polyethylene cup relative to the x-axis. In the radiological controls of the last follow-up examination, the presence of radiolucent lines around the trabecular metal augments as well as the incorporation of the graft were evaluated [23] (Fig. 2), although the latter was complicated by the placement of the mesh.

Radiographic loosening was defined by a change in the abduction angle of more than 10° or a change in the vertical or horizontal position of the acetabular component of more than 5 mm.

Data were analysed in SPSS version 18 for Windows. The data were grouped according to frequency and to measures of central tendency and dispersion: percentages, means, and standard deviation. To compare the variables under study with respect to time (before and after surgery), the nonparametric Wilcoxon test of ranks and signs for two related samples was used. Significance was accepted when the probability p associated with the test statistic was < 0.05.

For the WOMAC scale, the results were considered clinically relevant when the difference in the before–after evaluation exceeded predetermined values for each considered sphere, taking as reference 50% of the initial value [24].

## Results

The five patients had three 3A and two 3B bone defects according to the Paprosky classification and two IV and three III defects according to the Saleh classification. In every case, a large mesh was used in the posterosuperior area. In three cases, a ring mesh was used on the bottom of the acetabulum.

As the augments, we used two 58/20 (case s1 and 2), one 66/20 (case 3), one 62/20 (case 4), and one 54/20 (case 5). These sizes give an idea of the magnitude of the bone defects.

### Clinical results

**Fig. 3:**
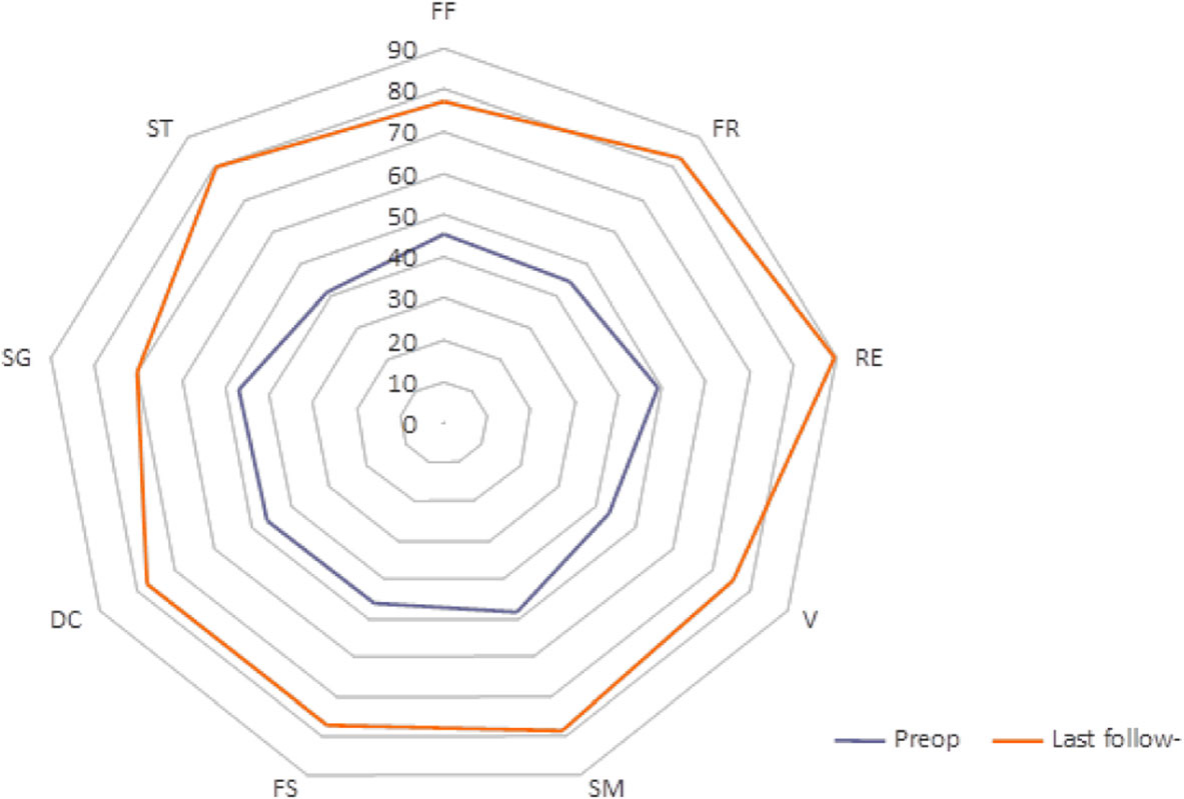
Radar graph of SF-36 score: physical functioning (PF); role physical (RF); role emotional (RE); vitality (V); mental health (MH); social functioning (SF); bodily pain (BP); general health (GH); physical component score (TS)

WOMAC pain, function, and stiffness improved for every patient at the last follow-up (Table 2). The results were clinically relevant and statistically significant (*p* < 0.05). The clinical results as shown by all components of the SF-36 improved at the last follow-up by clinically relevant and statistically significant degrees (*p* < 0.05) (Fig. 3).

**Table 2:**
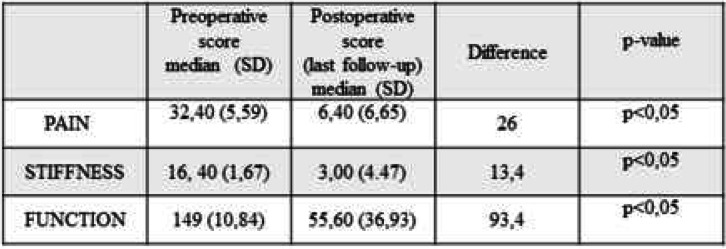
WOMAC scores

All the patients were satisfied with the result, and all would undergo the surgery again. It is noteworthy that patient 3, despite having experienced great improvement in the quality of life after the surgery, had WOMAC and SF-36 scores that were not very high. This is probably due to his individual comorbidities (rheumatoid arthritis, an important limiting factor in his daily activities).

### Radiographic results

Radiographs taken after surgery showed an improvement in the position of the new socket. Only case 3 showed a high hip centre (14 mm). In the other patients, the centre of rotation was lowered to its anatomical position. No significant differences were found in abduction angle (*p* = 0.27) or cup migration (*p* = 0.31) between the immediate postoperative radiograph and the last one. No patient had radiolucency at the bone-cement interface at the latest follow-up, and no patient had evidence of loosening around the augments.

Postoperative complications occurred in just one patient, case 5, a 50-year-old female who sustained two dislocations. The first episode was 6 weeks after surgery, and the second one 10 weeks after surgery. Both of them were treated with closed reduction. After discussing the different treatment alternatives with the patient, she preferred conservative treatment with a hip abduction orthosis. She has currently returned to her normal life, though suffering from certain restrictions due to instability fear.

## Discussion

Acetabular revision surgery, in cases of large acetabular defects, is a challenge for orthopaedic surgeons. Many papers have reported different results according to the type of reconstructive surgery. In general, there are two types of reconstruction. On the one hand, a highly porous-coated hemispherical cup with or without augments is associated with a cage in very large defects. These augments address bone loss and restore the centre of the hip [6]. On the other hand, there is biological reconstruction with bone impaction grafting. The advantage of this technique is that the bone stock is preserved and, possibly, restored for future revisions, which is particularly important for young patients [25], considering that hip prostheses are increasingly placed in younger patients, which gives an indication of the number of reviews that will be carried out [26].

The bone impaction grafting technique is well developed with good clinical results for contained defects or smaller segmental defects [27, 28]. The problem with this technique is in the large defects, that is, segmental defects with involvement of the acetabulum that affect more than 50%, which may be associated with involvement of the walls and acetabular columns. The results in such cases are disappointing [13, 14, 29]. Buttaro et al. [13] reported 23 uncontained acetabular defects. In all of them, there was migration of the acetabular components. Only two cases were revised for mechanical failure over a mean follow-up of 36 months. The problem with this study was the short time allowed for the follow-up of this reconstruction, because the mechanical failure rate would probably be greater in the longer term. Wilson et al. reported poor results, with a 30% failure rate, when using a large mesh in the posterosuperior area or a double mesh to reconstruct the defect. Similarly, Garcia- Rey et al. [14] published unsatisfactory results when treating Paprosky 3A and 3B defects, and they warned that another type of reconstruction could be needed in these cases. Furthermore, not all cases receive mesh on the posterosuperior part. In our opinion, practically all large defects (3A and 3B) require the placement of a mesh on the ceiling to make sure the defect is contained and to be able to perform impaction grafting. In these cases, when there is involvement of the wall, it is very difficult to obtain good fixation of the screws that hold the mesh in the wall and posterior column to achieve excellent mesh stability. This aspect is fundamental in reconstruction with impacted grafts. In turn, large defects require a significant volume of allograft, which can contribute to its resorption.

Thus, Schreurs et al., from the Nijmegen school, recognize that the outcome of impaction bone grafting will be less favourable in large defects, and its limitations still have to be defined [10]. Therefore, the poor results for large defects make it necessary to consider trabecular metal augments and thus the use of less bone allograft (fewer fresh-frozen bone chips). Knowing the inherent characteristics of trabecular metal, which provides excellent biological fixation and osseointegration [30], we considered the use of trabecular metal augments for severe combined deficiencies.

Few papers have described the combined use of trabecular metal augments with bone impaction grafting. To our understanding, only three papers have assessed the results of this type of reconstruction [22, 31, 32] .Borland et al. [22] included 24 hips with Paprosky 3A and 3B defects. The mean age was 62 years, and the follow-up was 61 months. There was one failure that underwent a new acetabular revision. In five cases, the migration of the polyethylene cup exceeded 5 mm. In none of the cases did they use a large mesh in the posterosuperior area to close the defect, and they mostly used augments. It is evident that this can be difficult to achieve in the largest defects, Paprosky 3B, which may explain the migration. Gill et al. [31], from Exeter, described 15 defects of Paprosky types 2B and 3A. The mean follow-up was 39 months, and the mean age was 68 years. Seven patients had previously undergone impaction grafting with a rim mesh. None of the patients had failure. It is noteworthy that most defects reported were small (1, 2A, and 2B), and the Exeter School, together with the Nijmegen School, developed the graft impaction technique [29, 33] (Table 3). The radiological assessment of migration is hard to interpret. The use of the mesh, and in our case, the use of TM augments, makes it difficult to assess the possible migration of the polyethylene cup. This radiological migration has also been studied through radiostereometric analysis. Ornstein et al. [34] published 21 defects of types I, II, and III according to the Gustilo classification, all but one of which had a migration of the polyethylene cup.

**Table 3:**
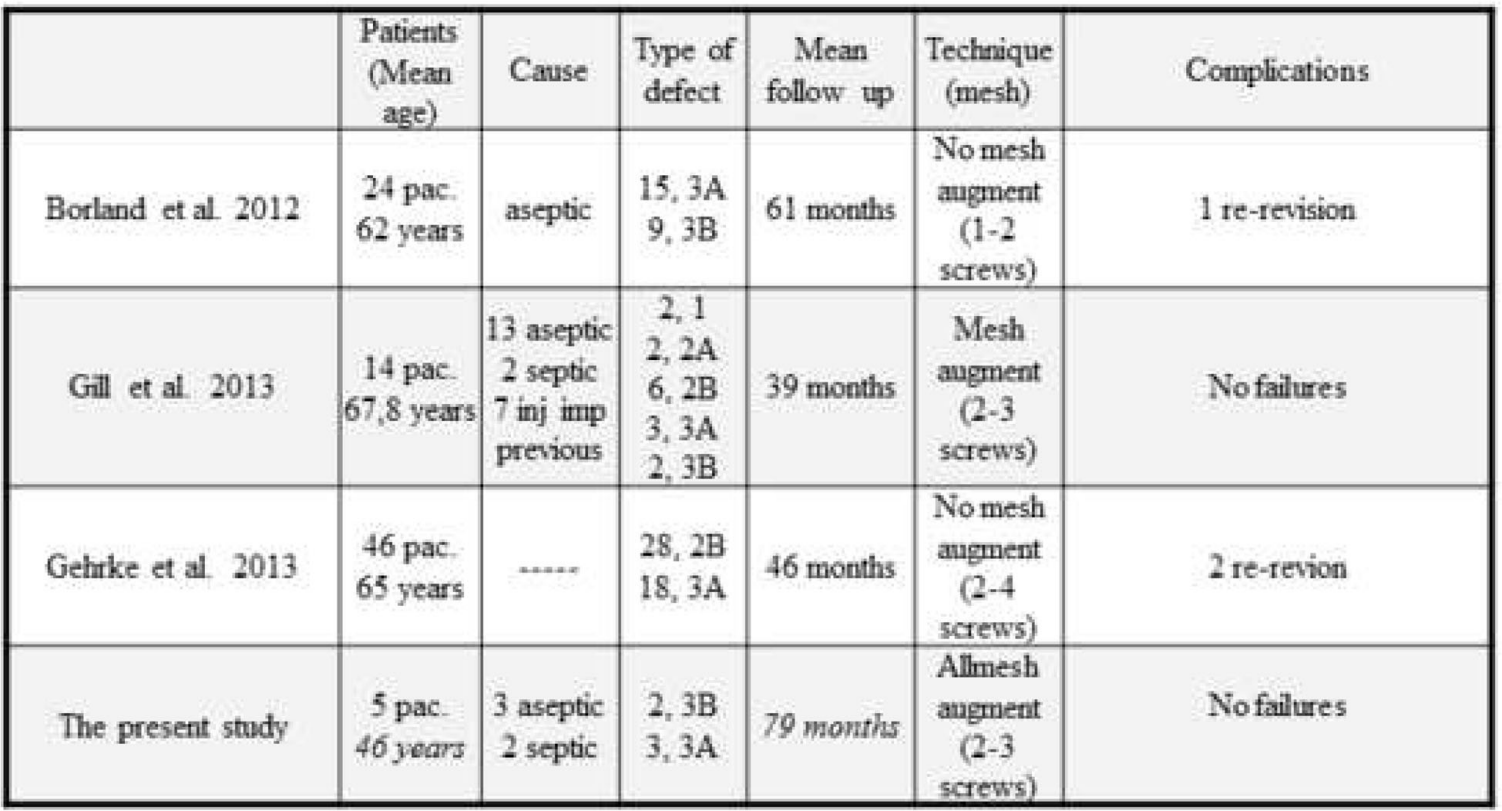
Published results of impaction grafting with trabecular metal augments for the treatment of uncontained acetabular defects

The results of our series are promising for large defects. We believe that to achieve a stable construction, it is essential that the augment be mostly in contact with the iliac bone, placing it in the most appropriate position for this to take place, regardless of whether the augment covers the defect. It is necessary to use at least two 6.5-mm screws to affix the augment.

The augment acts as a scaffolding for bone ingrowth and remodelling while providing load-bearing structural support [35]. The excellent results obtained with the augments are supported not only by the osteoconductive properties of this material [36] but also by its osteoinductive properties [37]. Another important advantage of tantalum is that there is no associated resorption, unlike allografts.

We used this type of reconstruction in two septic revisions, both in two stages. This reconstruction is controversial in septic cases. Rowan el al [38]., in a comparative study of reconstruction with trabecular metal vs. impaction grafting, points out that caution should be exercised with the latter in patients healing from infection. Trabecular metal can decrease the probability of infection and thus, from a clinical standpoint, is associated with a lower probability of infection [39], but this could not be demonstrated in in vitro experiments [40].

## Conclusions

Although the small number of cases is a limitation of the present study, we believe that the case series is very specific, as it comprises young patients, all aged 50 and under, about whom the large acetabular defect reconstruction literature is scarce. The study also has a long follow-up, which reinforces the results obtained. Currently, we still perform this type of reconstruction, increasing the size of the series but with shorter follow-up times.

When dealing with large defects in young patients, bone loss must be restored. Therefore, according to our results, the acetabular reconstruction technique combining trabecular metal augments with bone impaction grafting must be considered for large defects in young patients.

## Supplementary information

**Additional file 1.**

## Data Availability

The data used to support the findings of the present study are available from the corresponding author upon request.
