# Supplementary material for: Bone impaction grafting with trabecular metal augments in large defects in young patients: unravelling a new perspective in surgical technique
Source: BMC Musculoskelet Disord. 2020 Aug 27;21:581. doi: 10.1186/s12891-020-03591-w (PMC7453526; doi:10.1186/s12891-020-03591-w)
Supplement: Supplementary file 1 — Additional file 1. [file 12891_2020_3591_MOESM1_ESM.doc]

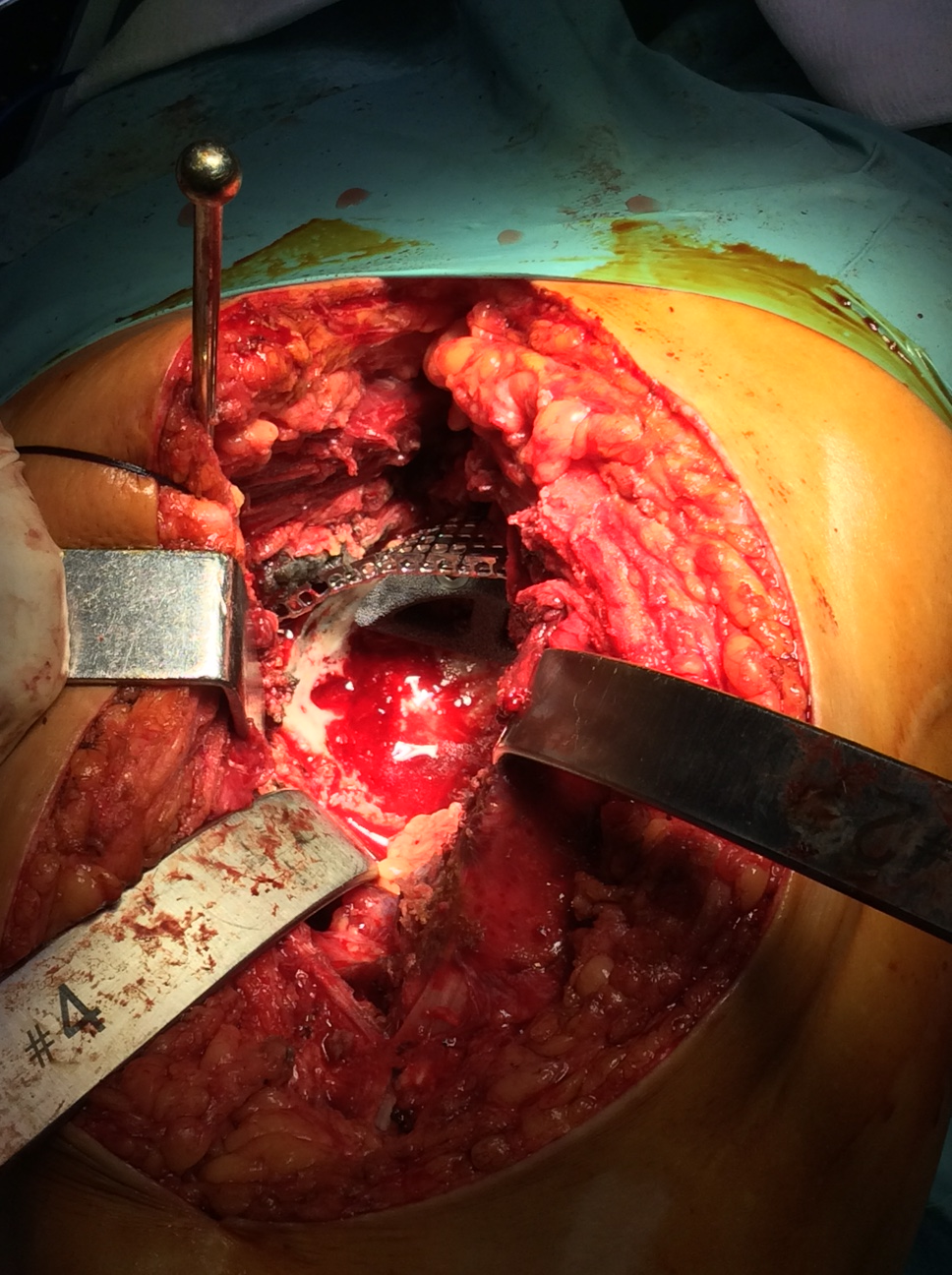

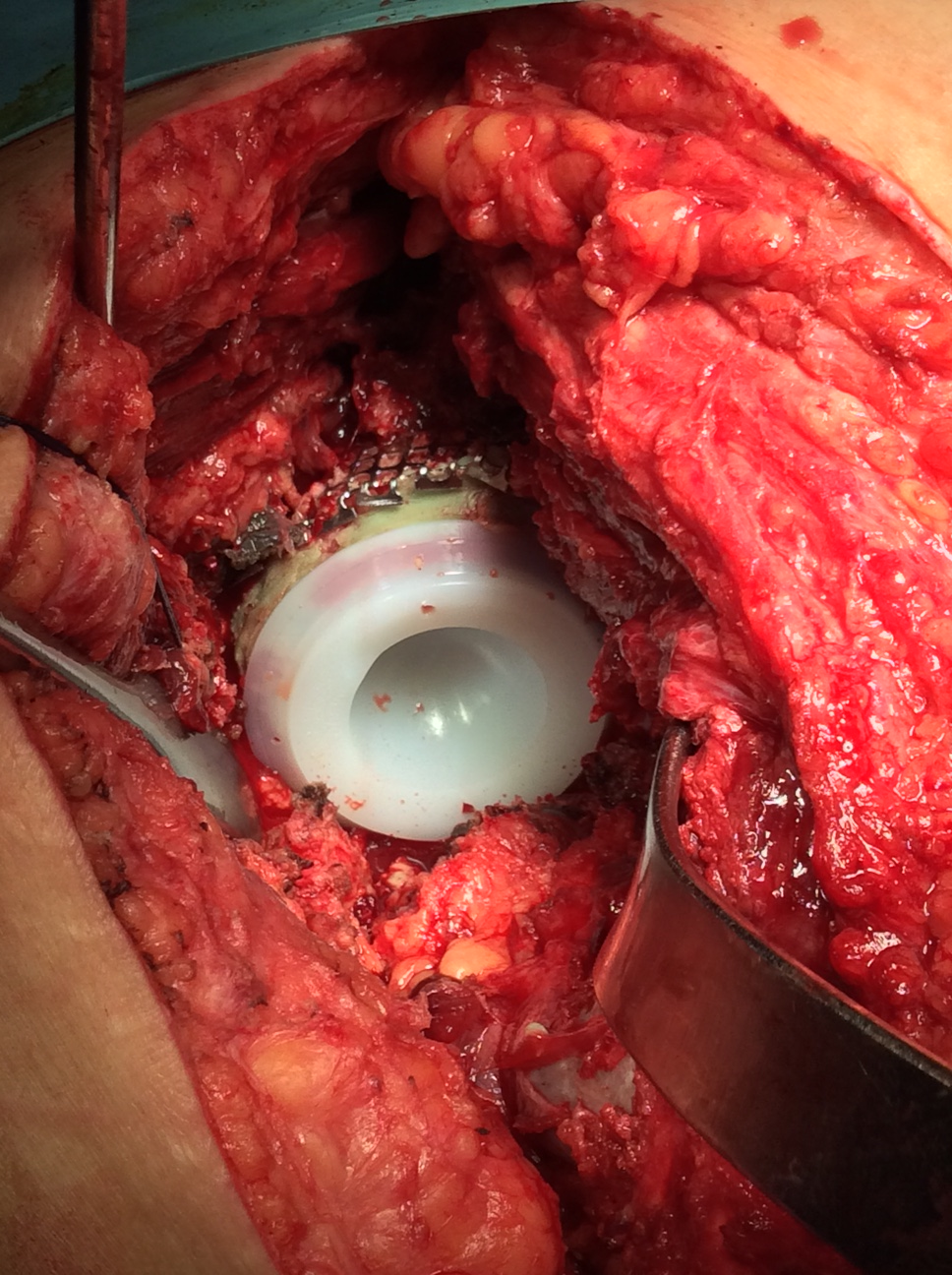


***Intraoperative image with the fixation of the tantalum augment with screws in combination with the large mesh in the posterolateral area of the acetabulum in a woman 50 years old (case5.)***


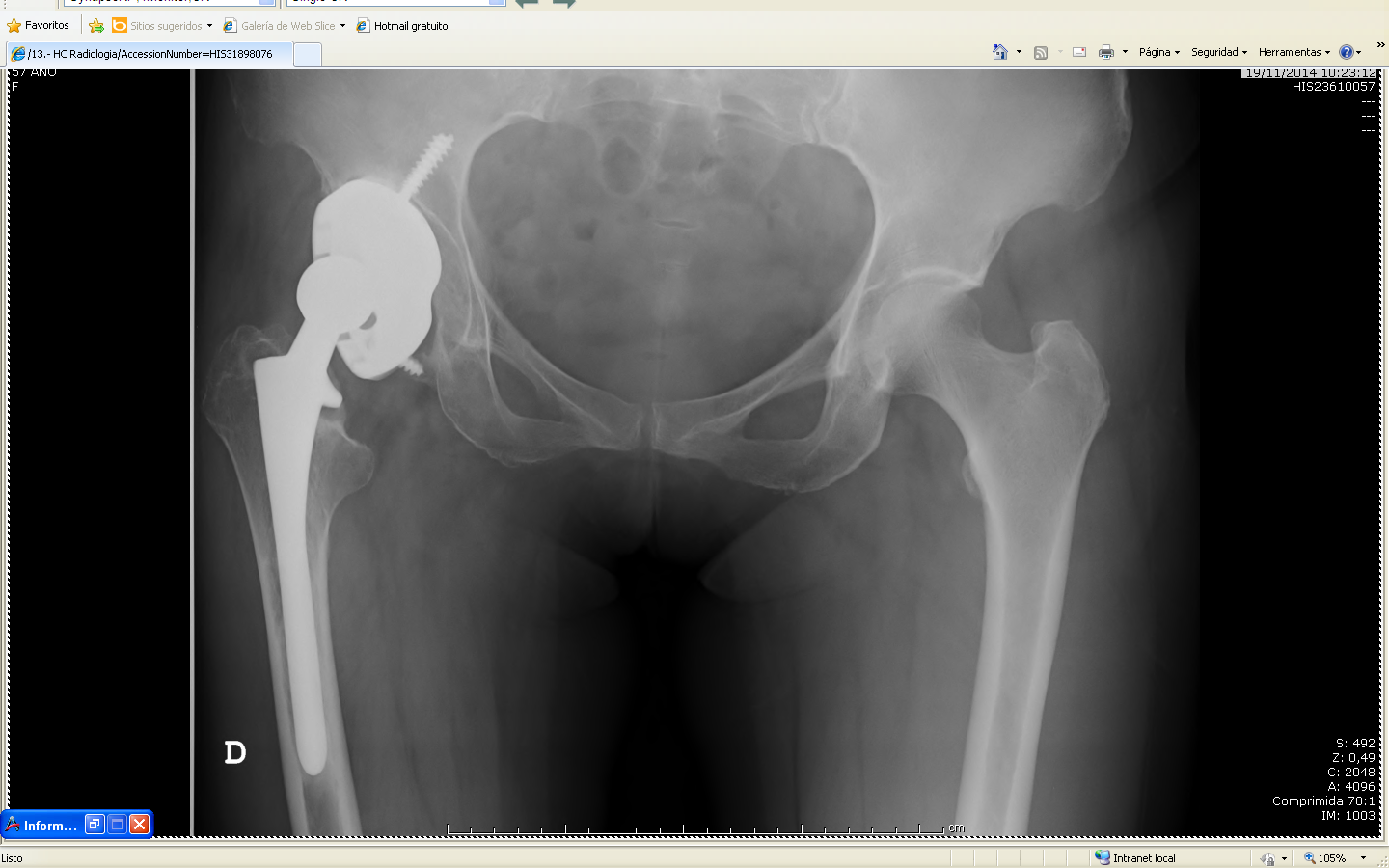

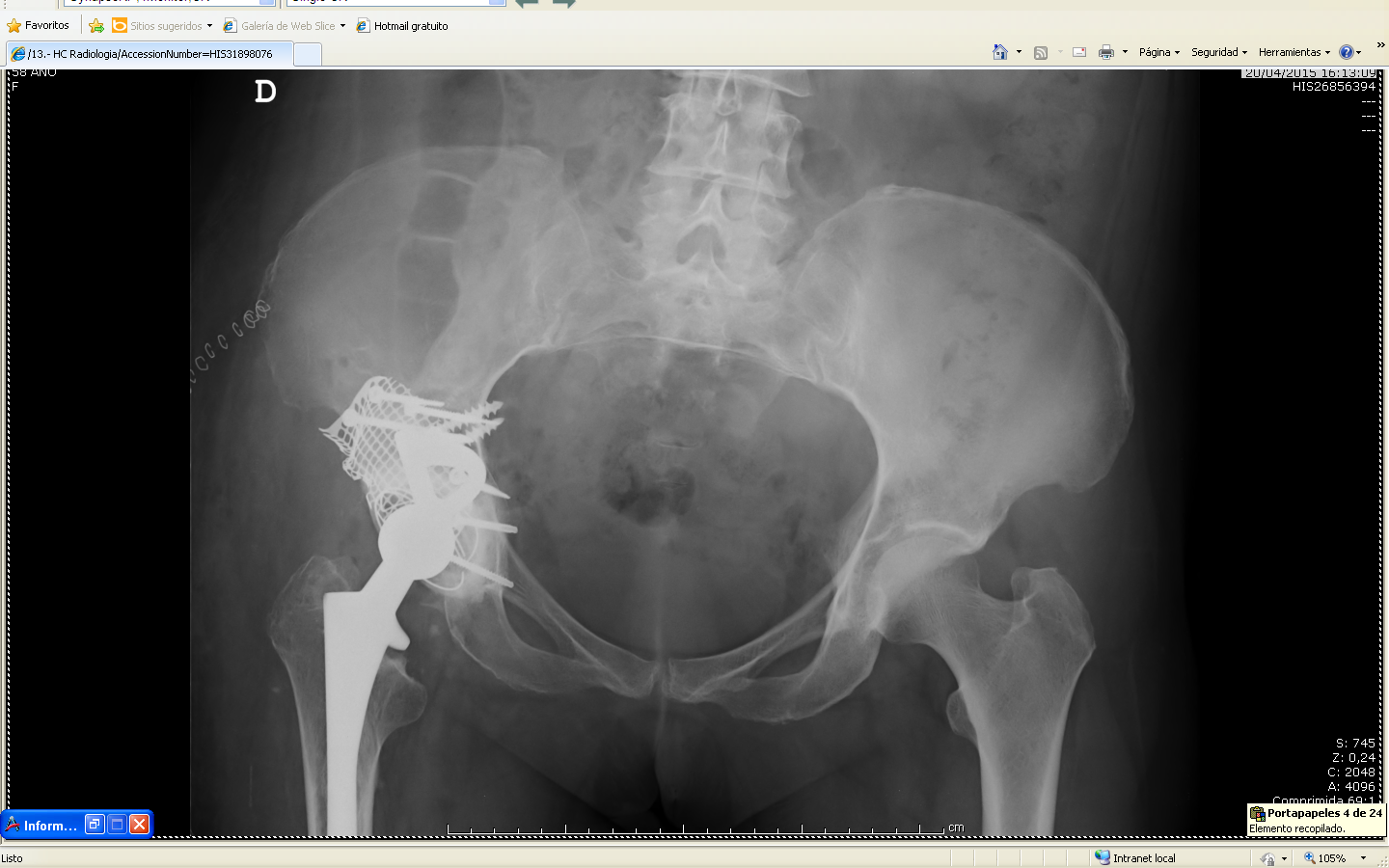


***Preoperative radiograph with aseptic cup loosening (case 5). Left side acetabular revision including tantalum-bone impaction grafting combination in immediately postop (case 5).***


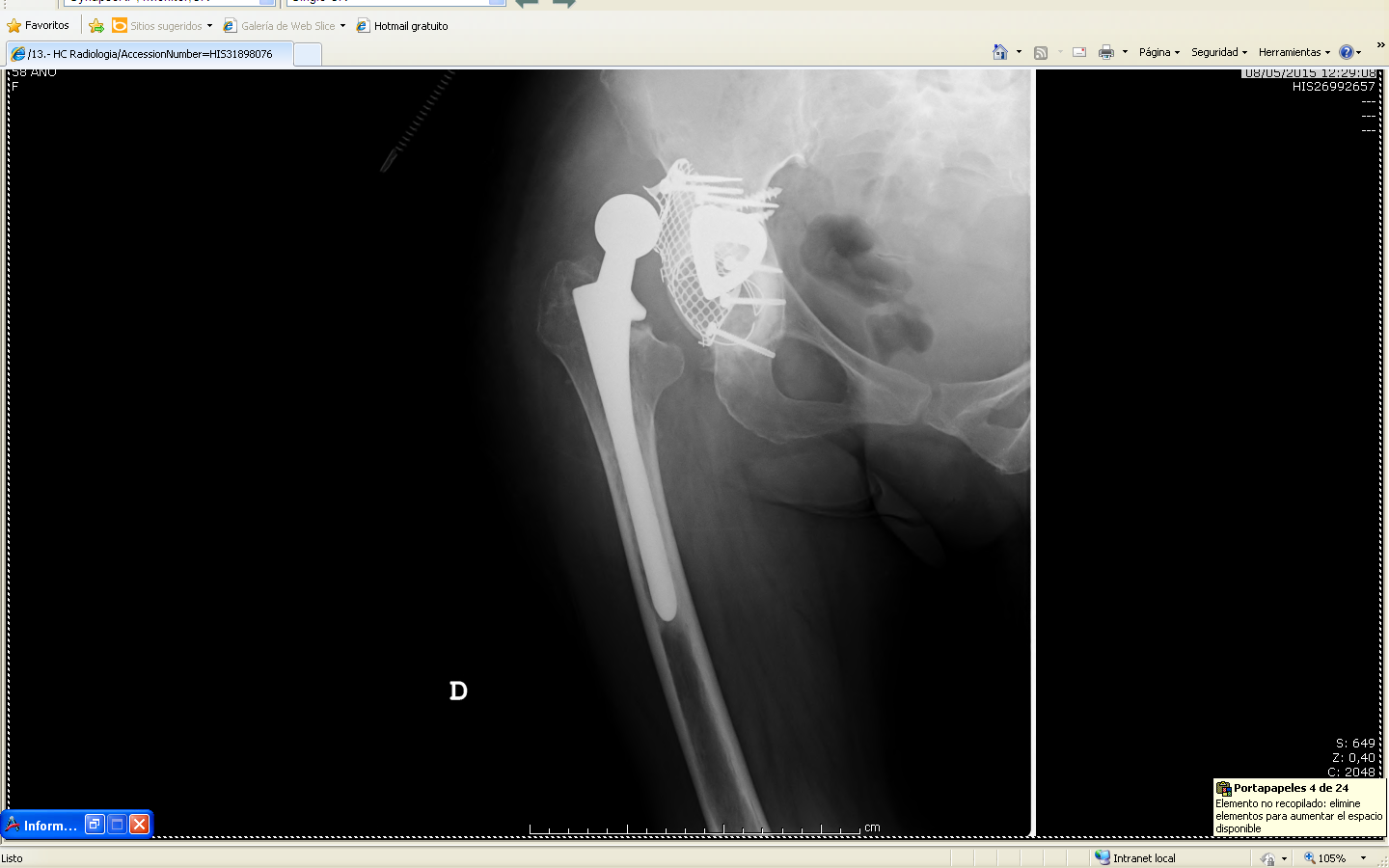

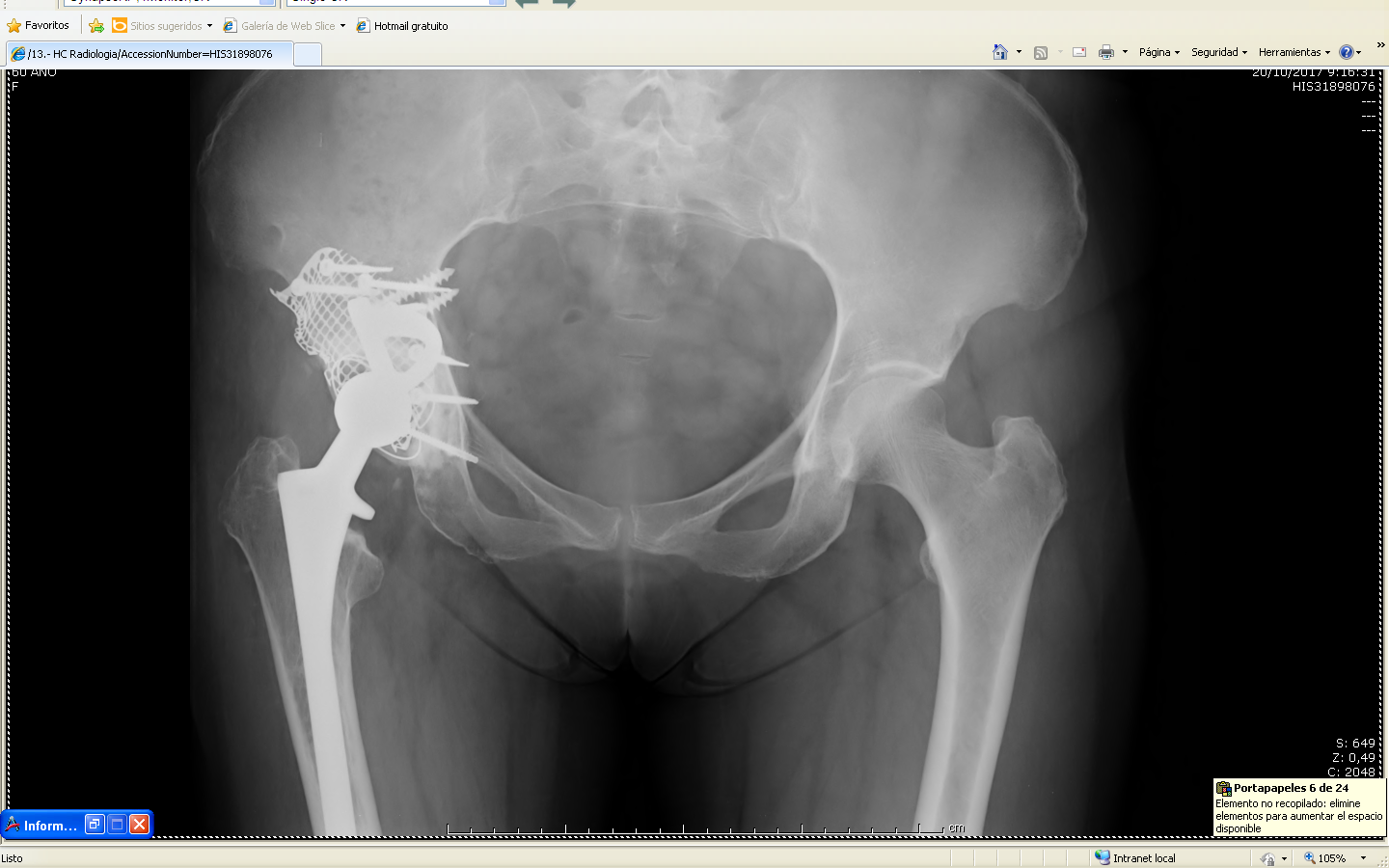


***Postoperative radiograph in his first dislocation episode 6 weeks after surgery. On the right side, the postoperative radiograph 60 months after surgery (case 5).***


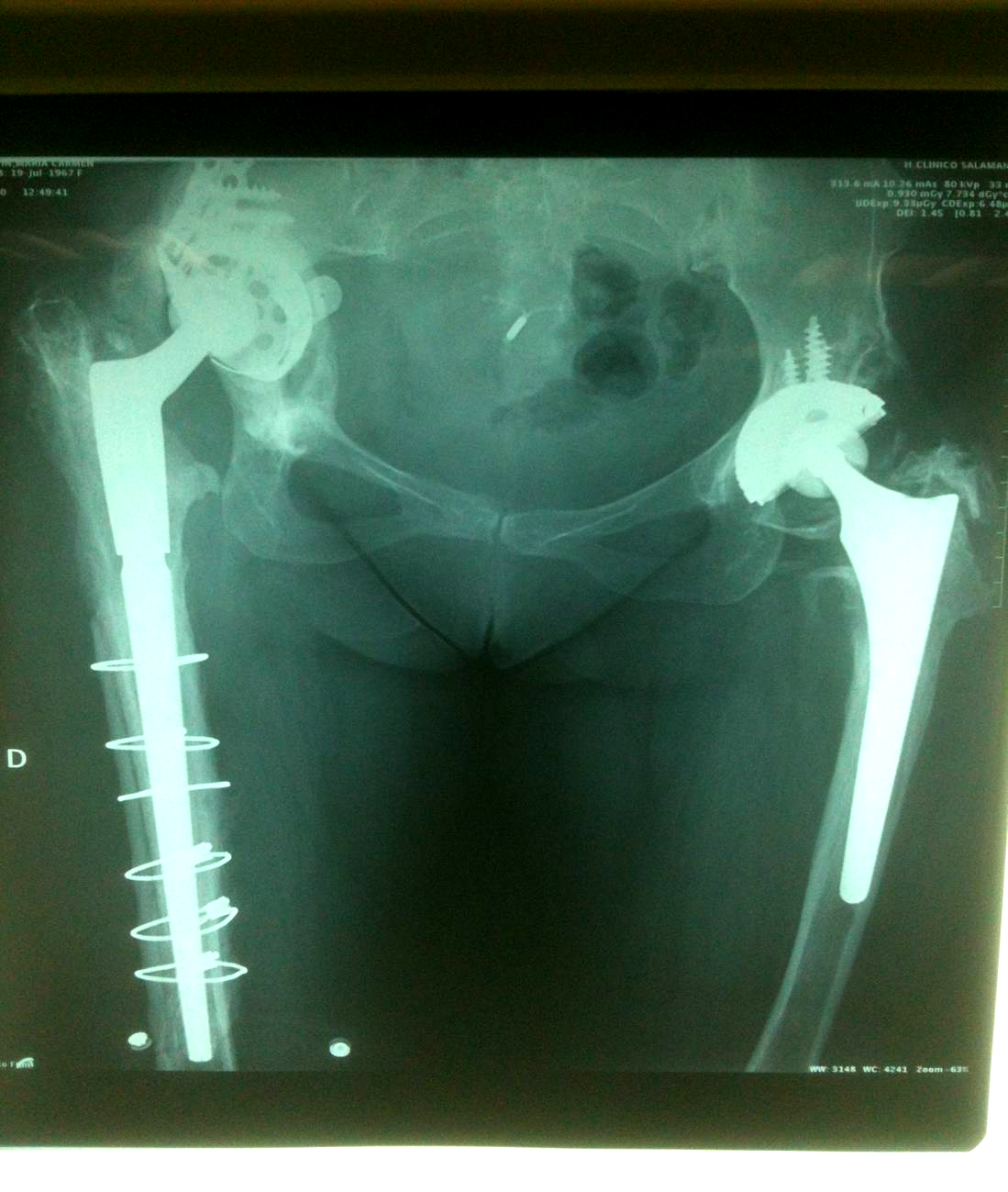


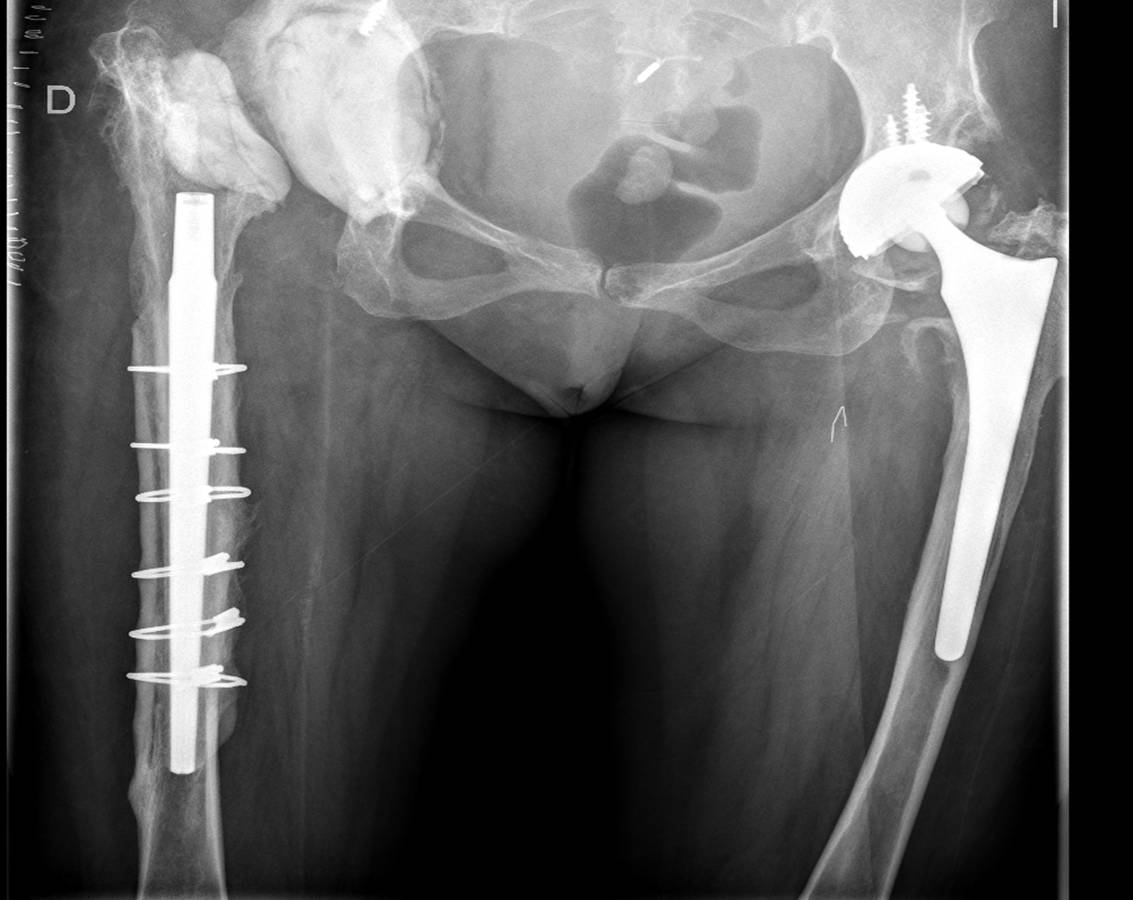


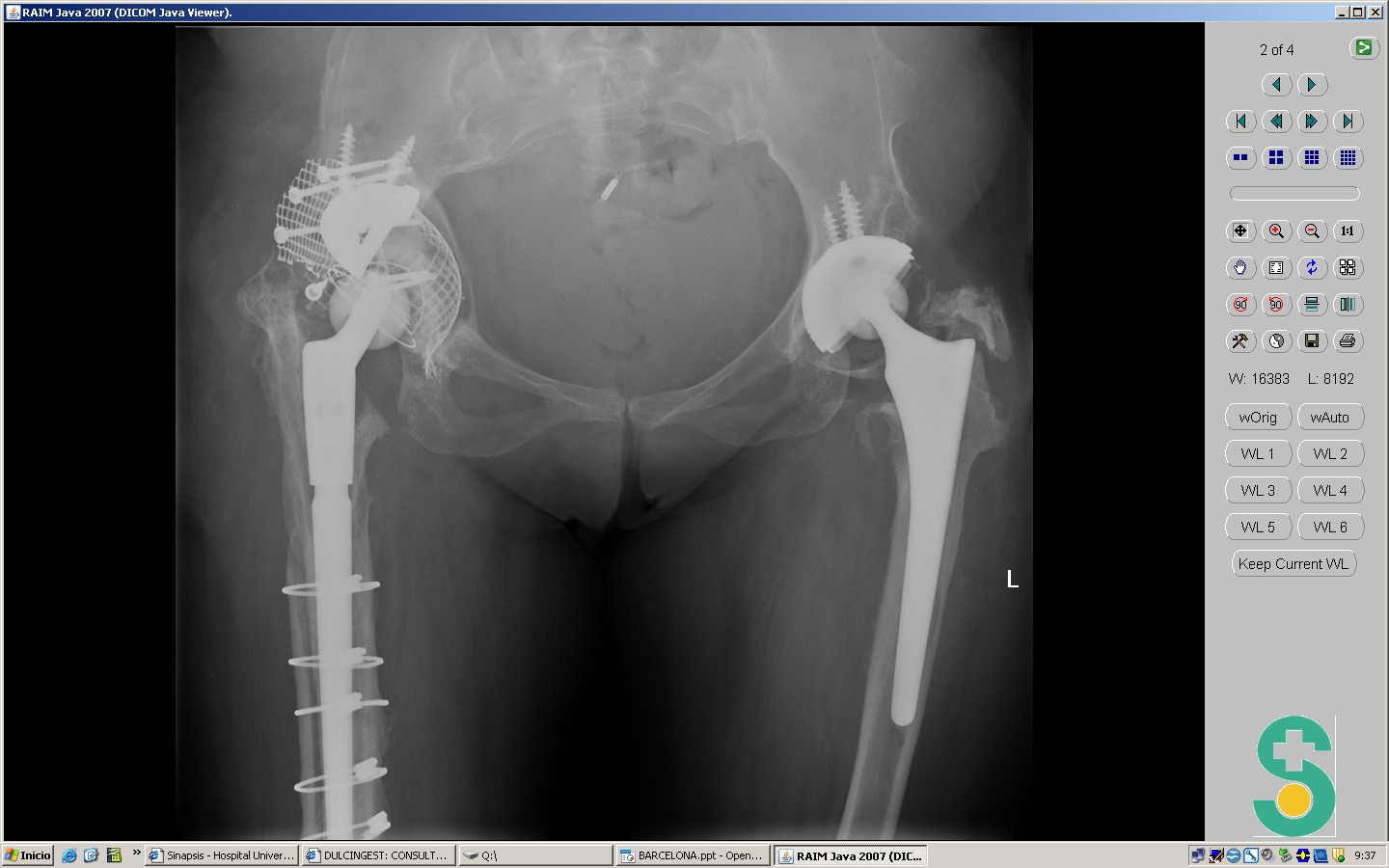

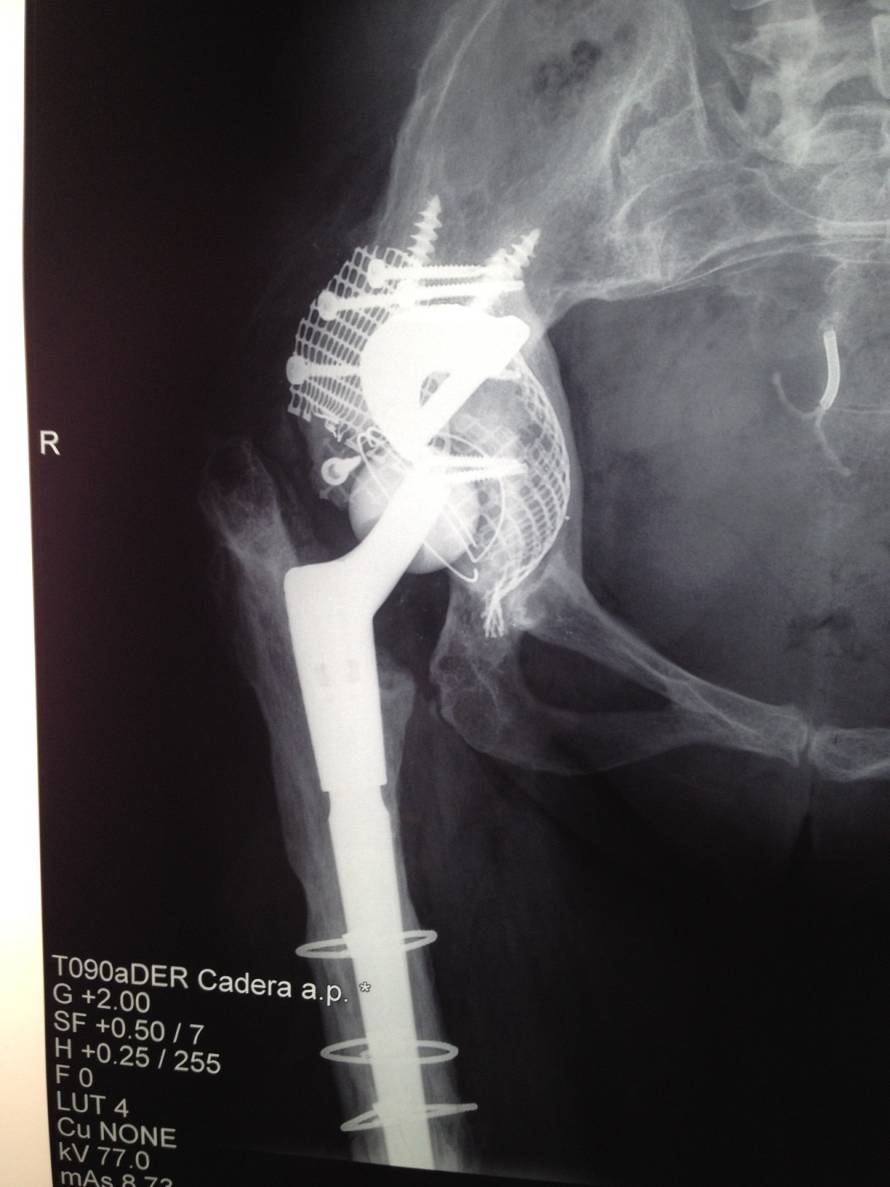


***Case 1: Women 45 years old with a septic loosening (S Epidermidis) of the left side. She was treated with a partial two stages revision. The reconstruction was done with trabecular metal and bone impaction grafting. The immediately postoperative radiograph on the left side, and radiograph 101 months after surgery.***
